# Conservative Approach of a Dentigerous Cyst

**DOI:** 10.1155/2021/5514923

**Published:** 2021-06-18

**Authors:** Farah Chouchene, Wassim Ben Ameur, Habib Hamdi, Maissa Bouenba, Fatma Masmoudi, Ahlem Baaziz, Fethi Maatouk, Hichem Ghedira

**Affiliations:** ^1^Pediatric and Preventive Dentistry Department, Faculty of Dental Medicine of Monastir, Laboratory of Biological, Clinical and Dento-Facial Approach, University of Monastir, Monastir, Tunisia; ^2^Dental Surgery Department, Faculty of Dental Medicine of Monastir, Monastir, Tunisia

## Abstract

Dentigerous cysts (DC) are the most common odontogenic cystic lesions of inflammatory origin occurring in children. These lesions can be treated by enucleation with or without related impacted teeth or marsupialization/decompression. The latter procedures have been used successfully for many years, but decompression is increasingly recommended in children because of its good outcomes and the preservation of the developing tooth. This conservative surgical technic allows simultaneously the normal eruption of the involved permanent teeth and the ossification of the bony defect. The present report describes an 8-year-old female patient with an inflammatory DC associated with an endodontically treated primary molar accidentally discovered on panoramic radiograph. Decompression of the cyst was performed, by means of a removable space maintainer acting as an acrylic obturator after removal of teeth 83 and 84. Ten months after the decompression procedure, a complete resolution and regression of the cystic lesion with full spontaneous eruption of the premolar were observed.

## 1. Introduction

From the age of 5 to 12 years, children are in mixed dentition. At this age, to ensure proper eruption of the permanent dentition and to prevent arch length deficit and loss of space, it is important to preserve the primary teeth [1].

Several disturbances can cause either infection or necrosis of the pulp tissue of primary teeth, and since pulp therapy is not always easy to perform on these teeth because of their complex anatomy, the preservation of primary teeth can induce undesirable effects on the permanent successor, such as the formation of inflammatory DC [2].

These cysts are caused by the accumulation of fluid between the reduced enamel epithelium and the crown of the tooth [3]. Although the canines and molars are the most affected, cystic formations involving the crown of the premolars and incisors are rare [4, 5].

Enucleation with or without impacted teeth or marsupialization/decompression are the treatments of choice of inflammatory DC. Both procedures, marsupialization and decompression, have been used successfully for many years, but decompression is increasingly recommended because of its successful outcomes and preservation of the developing tooth structure [6].

Since these cysts are more common in children, conservative management by decompression may be recommended, allowing the conservation of the permanent involved tooth [7, 8].

The present report is aimed at describing a complete regression of an inflammatory DC with full spontaneous eruption of the premolar ten months after a decompression procedure in an 8-year-old female patient.

## 2. Case Presentation

The present clinical case report was reported according to the CARE (CAse REport) Guidelines [9].

An eight-year-old female patient consulted the Department of Pediatric and Preventive Dentistry at the Faculty of Dental Medicine of Monastir for dental malocclusion management.

Written informed consent was obtained from the child's parents for all imaging exams, treatment modalities, and data publications.

**Figure 1:**
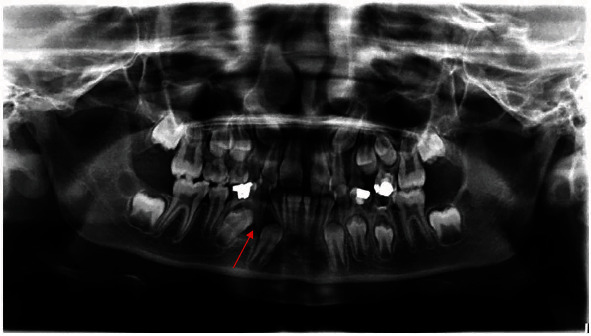
Preoperative panoramic radiograph (red arrow).

The panoramic radiograph performed for the interceptive orthodontic treatment revealed a large unilocular radiolucency repressing the dental germ of the first lower-right premolar (#44) including the lower-right deciduous canine (#83), and associated with a treated root of nonvital lower-right first primary molar (#84) (Figure 1).

The medical history of the patient did not reveal any specific systematic diseases or previous traumatic injuries in the affected area.

The extraoral examination seemed to show no abnormalities while the intraoral examination revealed an imperceptible expansion of the buccal cortical on the alveolar ridge in relation to teeth 83 and 84.

**Figure 2:**
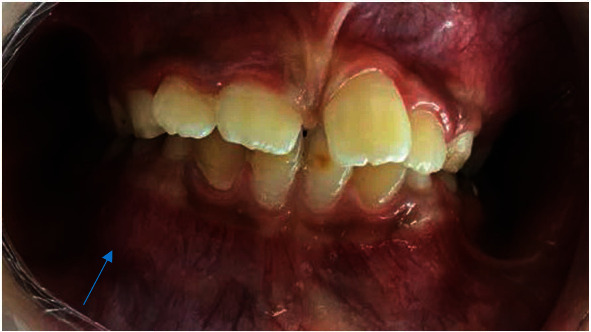
Intraoral view showing buccal hard swelling (blue arrow).

Inspection showed a normal looking mucosa (Figure 2). Palpation was painless with boney consistence. There was no motor or/and sensorial deficit at the orofacial structures.

**Figure 3:**
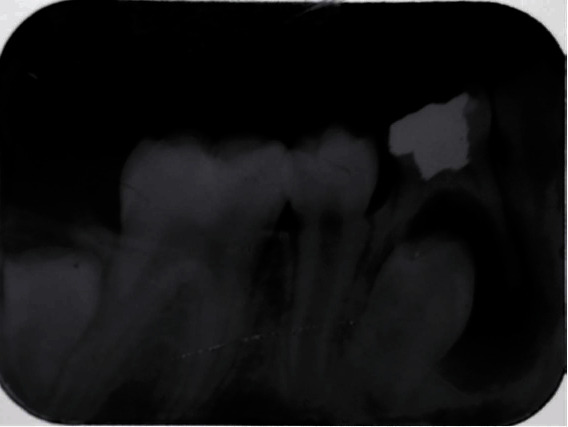
Retroalveolar radiograph showing radiolucency associated with an endodontically treated first right primary molar (tooth 84).

A retroalveolar radiograph was performed and showed a well-defined surrounded by a slight peripherical radiopaque thickening lesion apical to the lower-right first primary molar (#84) (Figure 3).

To confirm the lesion limits, a cone-beam computed tomography (CBCT) was requested.

**Figure 4:**
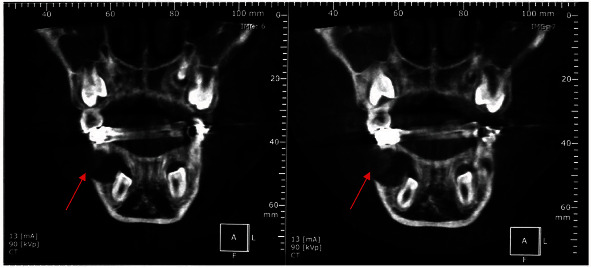
Sagittal sections showing a 12 mm diameter well-defined radiolucent lesion surrounding the crown of the unerupted first premolar (# 44) (red arrow).

**Figure 5:**
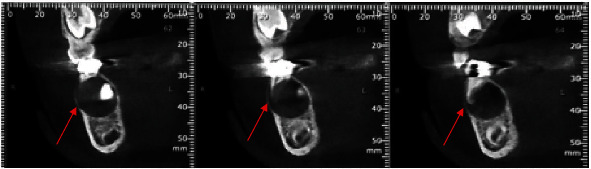
Coronal sections showing a well-defined radiolucent lesion surrounding the crown of the unerupted first premolar (# 44) (red arrow).

The sagittal and coronal views of the CBCT revealed a 12 mm diameter well-demarcated unilocular radiolucency (Figures 4 and 5).

The lesion was identified in the mandibular right region causing expansion of the buccal cortical with no signs of root resorption in the adjacent tooth and surrounding the crown of the unerupted first premolar (tooth 44).

The first lower-right premolar germ was forced against the lingual cortical with a mesial angulation. Based on the above-mentioned clinical and radiological findings, a provisional diagnosis of inflammatory DC was made.

The second primary molar and canine were extracted under local anesthesia, and a conservative approach by decompression to preserve the mandibular first premolar was followed.

**Figure 6:**
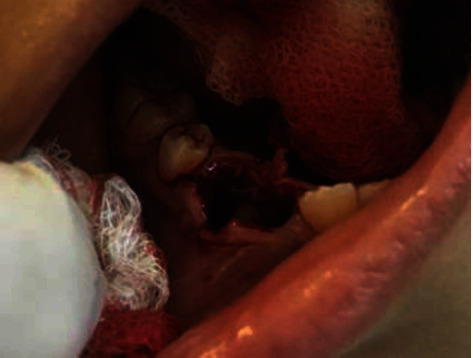
Decompression of the cyst and inspection of the right first premolar (tooth 44).

The extraction socket was extended to establish a communication between the cyst and the oral cavities without disturbing the erupting premolar (Figure 6).

The cystic fluid was evacuated under irrigation with normal saline solution through the socket.

An iodoform gauze was then packet into the lesion cavity, and sutures were placed.

The patient and her parents were advised to follow postsurgical instructions, rinsing the opening cyst twice a day with saline solution.

For the first 48 hours, postoperative care included the use of a cold pack. For pain management, 200 mg dexibuprofen three times a day was prescribed for the young patient.

**Figure 7:**
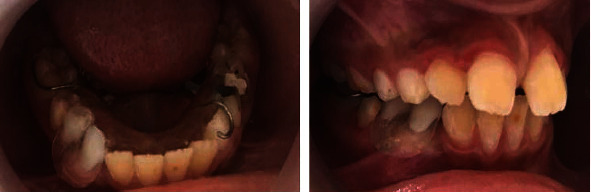
Removable space maintainer acting as an acrylic obturator used to guide the eruption of the premolar.

Two days postoperatively, an impression was made before gently removing the gauze which helped to prevent the alginate from getting the bone cavity. Then, a removable space maintainer acting as an acrylic obturator was designed (Figure 7).

During the surgical procedure, a tissue sample was taken for biopsy. Microscopically, the cyst was lined with cuboidal nonkeratinized stratified epithelium resembling reduced enamel epithelium, and the underlying connective tissue capsule showed chronic inflammatory cell infiltration. The histopathological examination confirmed the initial diagnosis of the inflammatory DC.

The acrylic obturator remained until the eruption of tooth 44 was visualized using a subsequent radiographic evaluation.

The follow-up appointments were scheduled every three months postsurgery.

**Figure 8:**
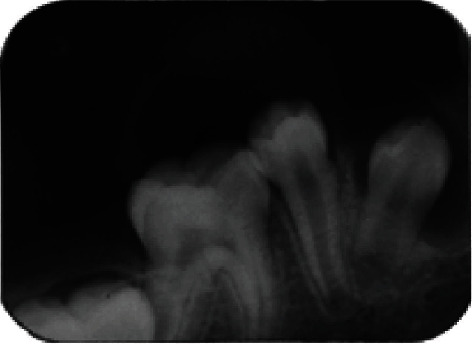
Three-month postoperative radiograph.

The three-month postoperative radiograph showed a reduction in radiolucency associated with a gradual spontaneous eruption of the tooth (Figure 8).

After 10 months follow-up, the radiographic and clinical findings showed that the affected teeth successfully erupted without any intervention.

**Figure 9:**
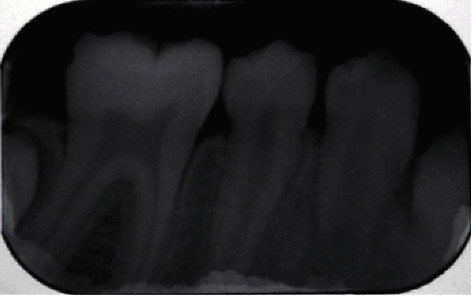
Twelve-month postoperative radiograph showing spontaneous eruption of the tooth 44.

**Figure 10:**
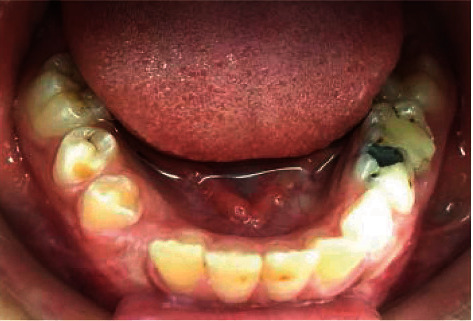
Intraoral view showing complete eruption of tooth 44 after 12 months follow-up.

The tooth was vital with almost a complete root formation (Figures 9 and 10).

## 3. Discussion

According to the World Health Organization (WHO) classification of odontogenic lesions, odontogenic cysts were divided into developmental and inflammatory cysts. The inflammatory odontogenic cysts included radicular cysts and inflammatory collateral cysts while the developmental odontogenic and nonodontogenic cysts included dentigerous cysts, lateral periodontal cysts, botryoid odontogenic cysts, gingival cysts, glandular odontogenic cysts, orthokeratinized odontogenic cysts, and nasopalatine duct cysts [10].

The DCs constitute the most frequent maxillary cystic lesions (30% of the odontogenic cysts) [11], with sometimes important local consequences that can cause dental shifts and root resorptions and even interrupt dental eruptions in the child. Therefore, early intervention is needed [12].

These cysts are also very common in mixed dentition and are usually associated with the roots of a nonvital or necrotic primary tooth and the crown of an unerupted permanent tooth [3], such described in the present report.

The pathogenesis of DC is still controversial. However, three possible mechanisms were proposed in the literature [4].

The first mechanism suggested that the developmental DC might form a dental follicle and might become inflamed later [4]. The second mechanism suggested that the formation of DC of extrafollicular origin may occur following the formation of a radicular cyst at an apex of a nonvital primary tooth followed by eruption of its permanent successor [4].

The follicle of permanent successor might get secondarily infected from either periapical inflammation of a nonvital primary tooth or other source leading to a DC formation, and this inflammatory process may explain the DC development in the present report [4].

For this reason, especially in mixed dentition, it is very important to establish not only a clinical but also a radiological follow-up of the endodontically treated primary molars until the eruption of permanent successors to be able to detect as soon as possible the side effects of pulp therapy and to manage them in prompt time.

Usually, DCs are asymptomatic and are discovered during routine dental radiographies [13].

Radiographically, these developmental cysts are characterized by the presence of a radiolucent, well-defined, unilocular lesion around the crown of an unerupted permanent tooth. They can be reported in the presence of exformation of the maxillaries, dental structures shifting, or delays in dental eruptions [14]. In the present case, the cyst lesion was discovered accidentally after a routine panoramic radiograph.

According to the literature, the DC management is exclusively surgical, and two treatment techniques have been suggested: the enucleation or the marsupialization/decompression.

In their retrospective study, Iatrou et al. choose the technique of enucleation, reserving the marsupialization to large cysts. An association of both techniques can be suggested for the more aggressive cysts to reduce the volume of the lesion, before surgical removal [15].

Conservative techniques for the treatment of odontogenic cysts have evolved gradually the last decades.

Marsupialization and decompression are minimal surgical intervention but decompression is less invasive because it requires a smaller bony window.

Decompression may envelop marsupialization, and it includes any method that keeps an opening into the exterior by using a conduit to decrease intracystic pressure [16].

The benefits of decompression are maintenance of pulp vitality, prevention of maxillary fractures, preservation of the inferior alveolar nerve or maxillary sinus, and low risk of recurrence [16].

In the present reported case, since the patient and her parents were cooperative, the decompression was recommended.

After decompression, the cystic cavity must remain open; numerous means have been described such as stents, stents fixed by mini-screws, packs of iodoform gauze, tubes for decompression, and retainers [17].

Ghandour et al. recommended the placement of a gauze during the first two days after the surgery to keep the surgical site open [18]. Preserving the fenestration open may allow to reduce the size of the cystic lesion postoperatively; to reduce the infection, iodoform can be used [19].

In mixed dentition, a loss of space may occur during the first three weeks following the extraction of the primary teeth; for this reason, maintaining the space is necessary to avoid the decrease in the arch length [20]. In the reported case, a removable partial denture was indicated both to keep the cyst cavity opening and intracystic pressure at a reduced level and to keep the space of the first premolar while guiding its eruption.

A clinical and radiographic follow-up will be necessary, every three months initially, then every six months, until the end of the eruption of the tooth and the healing of the bone [15].

Both radiological and clinical findings after 10 months showed in the present report that the first premolar had successfully erupted without any orthodontic intervention.

Although the predictive criteria for this eruption remain unknown, the size of the cystic lesion does not constitute a risk factor for the eruption.

In fact, the most favorable predictive factors are essentially the age of the patient (less than ten years old), the depth of the inclusion (less is better), and the angulation of the germ (inferior to 25°) [21].

After minimal surgical intervention, the spontaneous eruption may occur in approximately 70% to 90 of cases. The length of the eruption is very variable, ranging from seven months to five years, and orthodontic traction can be associated [15, 21].

## 4. Conclusion

Extraction of the primary infected tooth and decompression is the treatment of choice for infected dentigerous cyst in mixed dentition.

The surgical procedure can be followed by wearing of an acrylic resin obturator, incorporating features of space maintainer especially when primary molars are involved, both to maintain the surgical opening during healing and to prevent the arch length deficiency.

This report highlights the interest of performing a systematic radiological examination during the child's first visit.

## Data Availability

All data generated and analysed which related this case report are included in this published article.
